# Enhanced In Vivo Wound Healing Efficacy of a Novel Hydrogel Loaded with Copper (II) Schiff Base Quinoline Complex (CuSQ) Solid Lipid Nanoparticles

**DOI:** 10.3390/ph15080978

**Published:** 2022-08-08

**Authors:** Doaa Abou El-ezz, Laila H. Abdel-Rahman, Badriah Saad Al-Farhan, Dalia A. Mostafa, Eman G. Ayad, Maram T. Basha, Mahmoud Abdelaziz, Ehab M. Abdalla

**Affiliations:** 1 Pharmacology Department, Faculty of Pharmacy, October University of Modern Sciences and Arts (MSA University), Cairo 12566, Egypt; 2 Chemistry Department, Faculty of Science, Sohag University, Sohag 82534, Egypt; 3 Chemistry Department, Faculty of Girls for Science, King Khalid University, Abha 61421, Saudi Arabia; 4 Pharmaceutics Department, Faculty of Pharmacy, October University of Modern Sciences and Arts (MSA University), Cairo 12566, Egypt; 5 Chemistry Department, Faculty of Science, Helwan University, Cairo 11795, Egypt; 6 Department of Chemistry, College of Science, University of Jeddah, Jeddah 21589, Saudi Arabia; 7 Chemistry Department, Faculty of Science, New Valley University, Alkharga 72511, Egypt

**Keywords:** wound healing, copper Schiff base, hydrogel, solid lipid nanoparticles, 8-hydroxy quinoline

## Abstract

Wound dressings created using nanotechnology are known as suitable substrates to speed up the healing of both acute and chronic wounds. Therapeutic substances can be delivered using these materials. In this study, a hydrogel loaded with Cu (II) Schiff base 8-hydroxy quinoline complex (CuSQ) solid lipid nanoparticles (SLN) was formulated to investigate its wound healing potential in an excision wound healing model in rats. The CuSQ SLN were spherical shaped with sizes ranging from 111 to 202 nm and a polydispersity index (PDI) ranging from 0.43 to 0.76, encapsulation efficiency (EE) % between 85 and 88, and zeta potential (ZP) of −11.8 to −40 mV. The formulated hydrogel showed good homogeneity, good stability, and a pH of 6.4 which indicates no skin irritation and had no cytotoxicity on the human skin fibroblast (HSF) cell line. In the in vivo study, animals were placed in five groups: control, standard, plain hydrogel, low dose, and high dose of CuSQ hydrogel. Both doses of CuSQ showed significantly faster healing rates compared to standard and control rats. In addition, the histopathology study showed more collagen, improved angiogenesis, and intact re-epithelization with less inflammation. A significant increase in transforming growth factor-beta1 (TGF-β1) level and increased immune expression of vascular endothelial growth factor (VEGF) by CuSQ treatment validates its role in collagen synthesis, proliferation of fibroblasts and enhancement of angiogenesis. Matrix metalloproteinase-9 (MMP-9) was found to be significantly reduced after CuSQ treatment. Immunohistochemistry of tumor necrosis factor alpha (TNF-α) revealed a marked decrease in inflammation. Thus, we concluded that CuSQ would be a beneficial drug for cutaneous wound healing since it effectively accelerated wound healing through regulation of various cytokines and growth factors.

## 1. Introduction

Biopolymer-based wound dressings and bioengineered skin substitutes including hydrogels, sponges, and electrospun mats are generally thought to play a crucial role in wound healing as they can stop excessive water loss from the wound and protect it from infection [1,2]. Due to their resemblance to the extracellular matrix (ECM), hydrogels have been found to be particularly useful biomaterials for applications in tissue engineering and wound healing [3,4,5]. Benefits of hydrogels include proper absorption of wound exudate, adequate water vapor permeability, and provision of a moist substrate that promotes epithelium regeneration and patient compliance [6,7,8].

Recent studies have concentrated on the biological impacts of Schiff bases, especially their antimicrobial and anti-inflammatory properties. Schiff bases, which are generated when amine and ketones react, have a variety of biological effects, e.g., antioxidant capacity, analgesic, anti-inflammatory, and ability to restore hair and skin. Additionally, it has been indicated in various research that the chelation of Schiff bases with some metals, oxygen, and others, has been addressed to possess applications as broad therapies because of several biological processes [9]. Copper nanoparticles have been proven to play a key role in the facilitation of numerous enzyme activity and hence maintenance of many physiological processes in the body [10]. It is important to note that copper substituted Schiff bases are excellent anti-bacterial, anti-inflammatory, and antioxidant agents that work in a variety of ways to promote wound healing [9,11].

Recently, a Cu (II) Schiff base complex was developed to test the efficacy of using a new Cu (II) complex with a Schiff base obtained from bis [N-((5-chloro-1H-indole-3-yl) methylene) nicotine hydrazide] to treat wounds [12].

Due to the presence of oxygen and nitrogen atoms, the 8-hydroxy quinoline (Q) molecule has a strong chelating effect on metal ions. The Q compounds have a variety of pharmacologically advantageous effects, including antibacterial [13], antioxidant, anticancer, and anti-inflammatory effects [14].

It was noted that, the wound healing effect of copper (II) mixed ligand complex of Salen Schiff base ligand and 8-hydroxy quinoline is not much explored. By considering the above facts and in continuation of our previous investigations [9,15,16]; herein we report the synthesis, spectral characterization, in vitro antimicrobial, and in vivo wound healing evaluation of the Cu (II) mononuclear complex of an ONNO donor Schiff base ligand and 8-hydroxy quinoline.

## 2. Results

### 2.1. Physicochemical Properties

The CuSQ complex is colored and resistant to moisture and air. The analytical findings support the hypothesized chemical formula and demonstrate that 1:1:1 mixed ligand Salen/8-hydroxy quinoline complex was formed. The complex’s molar conductance in a 10^–3^ M DMF solution is 24.10 Ω^–1^cm^2^mol^–1^. This finding confirms the solution’s non-electrolytic nature (Table 1).

### 2.2. Fourier Transformation Infrared Spectra (FTIR)

**Figure 1:**
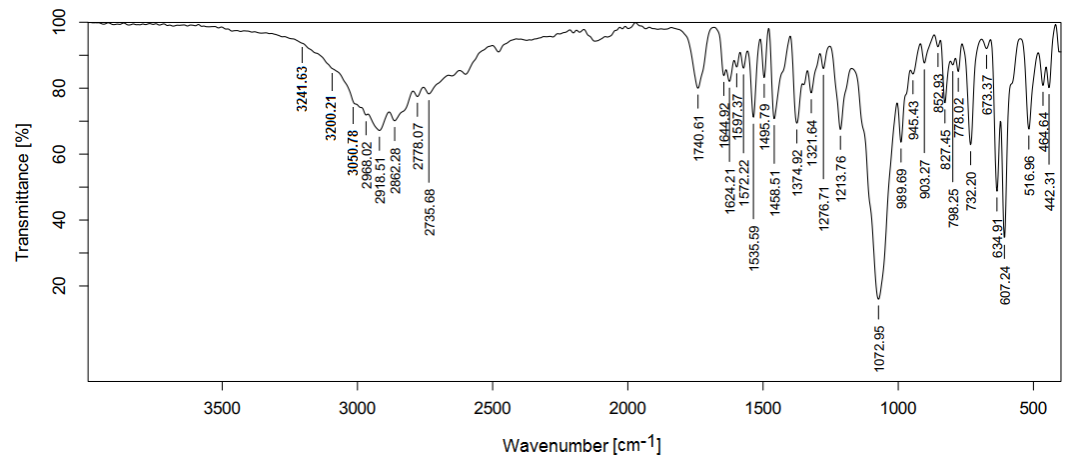
FT-IR spectrum of CuSQ complex in 4000–400 cm^–1^.

In Figure 1 and Table 2, the FTIR spectra of Salen and the CuSQ complex are depicted. A band at 1608 cm^−1^ in the spectrum of Salen may indicate the presence of the C=N az-omethine group. This band is moved to a higher frequency upon CuSQ complex synthesis (1624 cm^−1^). This demonstrates the azomethine nitrogen’s involvement in complex formation [17,18,19]. The shift of a band from 1247 cm^−1^ in Salen to 1213 cm^−1^ during complex formation due to the v (C-O) peak provides more evidence for the coordination of the phenolic oxygen. The formation of additional bands at 442 and 516 cm^−1^ that correspond to the stretching vibrations of the M-N and M-O bonds [20,21] provides support to this.

### 2.3. Electronic Spectra

**Figure 2:**
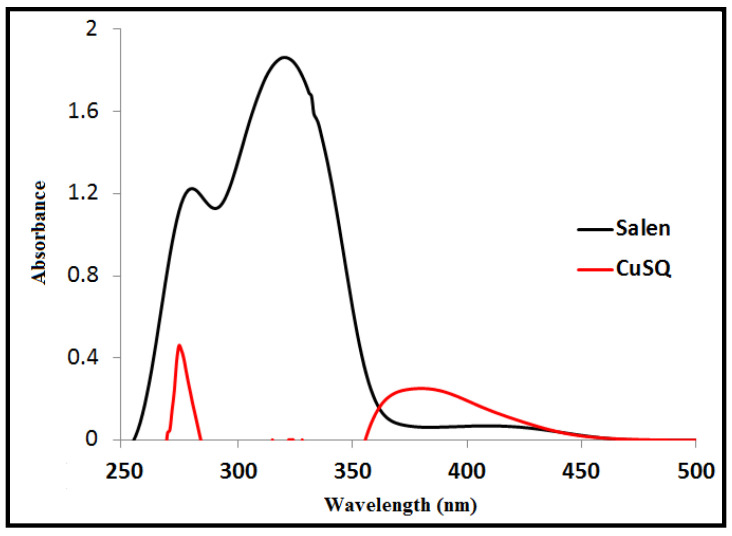
Electronic spectra of Salen ligand and its CuSQ complex in DMSO with concentration 10^−3^ M at 298 K.

Table 3 and Figure 2 display the UV-Vis spectra of the Salen ligand and its CuSQ complex in DMSO. Three absorption bands were observed in the Salen’s electronic spectra at wavelengths of 280, 320, and 409 nm. The π—π * transition was thought to be responsible for the first band at max = 280 nm. The second and third absorption bands, located at max = 320 and 409 nm, respectively, were attributed to the charge transfer and the *n*—π * transition of the (C=N) group. The coordination of the azomethine nitrogen to the Cu (II) ions was confirmed by the spectrum of the CuSQ complex, which showed that these bands are displaced to 280 nm and 380 nm for the π—π * and *n*—π * transitions, respectively [22].

### 2.4. Antimicrobial Bioassay

**Figure 3:**
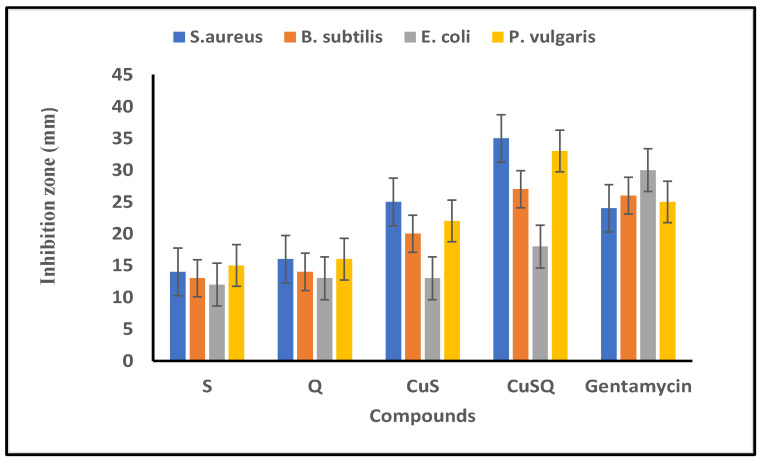
Antibacterial activity (inhibition zone) of Salen, CuS, S, Q, and CuSQ compared to gentamicin at concentration of 10 mg mL^–1^.

The results are listed in Table 4 and shown in Figure 3. These findings demonstrate that the CuSQ complex is a more effective inhibitor of microbial growth than the free Salen ligand. The inhibition zones for CuSQ are larger than those for gentamicin against *S. aureus* (+ve) (35 mm) and *P. vulgaris* (−ve) (33 mm), while the inhibition zones of gentamicin are (24 mm) and (25 mm) respectively. The inhibition potency of CuSQ against *B. Subtilis* (+ve) is superior to that of gentamicin, where the inhibition diameter is 27 mm while that of gentamicin is 26 mm.

### 2.5. Characterization of CuSQ-Loaded SLNs

#### 2.5.1. Scanning Electron Microscope (SEM)

**Figure 4:**
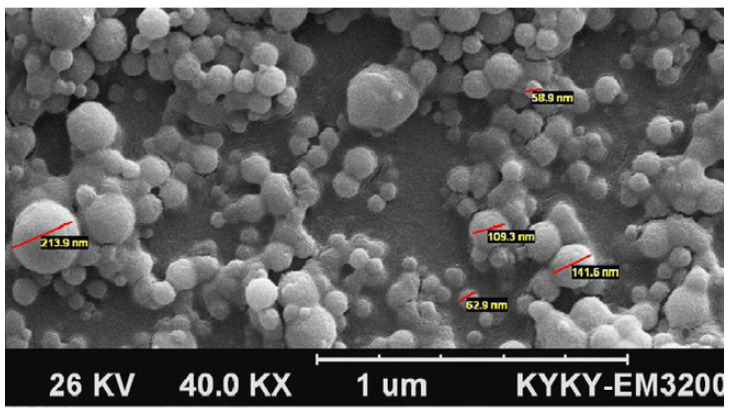
Scanning electron microscope (SEM) images of CuSQ-loaded solid lipid nanoparticles (SLNs).

The CuSQ-loaded SLNs were characterized by scanning electron microscope (SEM) as shown in Figure 4. Particles were smooth and spherical in shape. Additionally, due to the lipid nature of the carriers, the presence of surfactants, and sample preparation prior to SEM examination, particles typically clustered together.

#### 2.5.2. Particle Size, Polydispersity Index (PDI), and Zeta Potential Analysis

To confirm that the particles of the SLNs are in the nanoscale range, particle size measurement was carried out. All SLNs we prepared were in the nanoscale, with typical particle sizes ranging from 100 ± 0.26 nm to 202 ± 0.42 nm (*n* = 3). Moreover, all formulations had low values of polydispersity (0.43 ± 0.12–0.52 ± 0.11) (*n* = 3) which ensure particle size uniformities as shown in Table 5. Regarding zeta potential, values were −11.8 mv ± 0.13 and −40 mv ± 0.23 as shown in Table 5. Zeta potential measurement enables colloidal dispersion and storage stability predictions. Due to electrostatic repulsion, charged particles with a high zeta potential are less likely to aggregate.

#### 2.5.3. Entrapment Efficiency

The encapsulation efficiency percentage (EE%) was in the range between 85–88% as shown in Table 5. The proportion of core material incorporated into the nanostructure in relation to the overall amount of core material introduced during the encapsulation process is known as the EE%. EE is equal to (mE/mT) 100%; where mE is the mass of the core material integrated and mT is the total mass of the core material introduced.

### 2.6. Cytotoxicity Assay

Analysis of the cytotoxic effects of the CuSQ SLN on the viability of the HSF cell line using SRB assay was performed. The CuSQ SLN were non-cytotoxic and the IC_50_ was 1.2 μg/mL.

### 2.7. Evaluation of Hydrogel Physical Characteristic

The formulated hydrogels showed good homogeneity, good stability, and a pH of 6.4 which indicates that it is not irritant to skin.

### 2.8. In Vitro Drug Dissolution of Hydrogel

**Figure 5:**
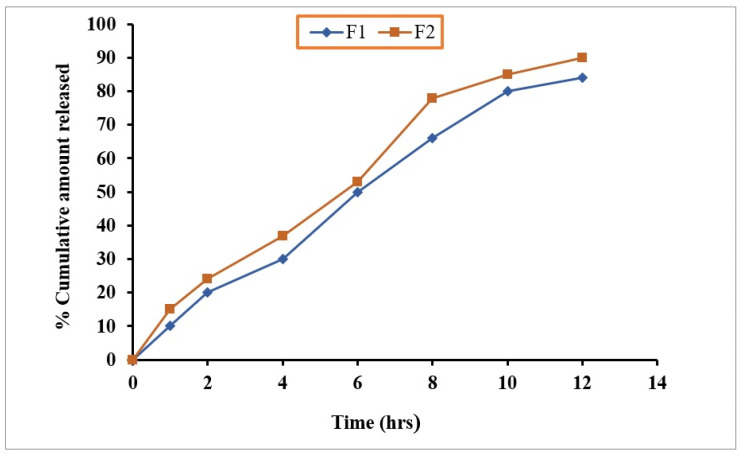
In vitro drug release of low dose (F1) and high dose (F2) of CuSQ hydrogel.

To determine the release profile, the cumulative released amount of CuSQ from the hydrogel was plotted against time. A rapid initial release was noted, which was followed by a slower release rate. The slower release in the later stage was attributed to the fact that solubilized drugs can only be released slowly from lipid matrices due to dissolution and diffusion. The first burst rate may be caused by drug desorption associated with the surface of nanoparticles. The drug could be transferred right away into the receiver compartment due to the dialysis membrane. The release study showed the sustained release of CuSQ over 12 h to reach 90% of drug release. The first hours up to 5 h showed 30–40% of released drug which confirmed that slightly prolonged drug release has occurred. The proper selection of lipids and surfactants can affect the release profile; in fact, the in vitro release from the SLN dispersion was found to be in the range of 40% to 90% at the end of 12 h (Figure 5).

### 2.9. Effect of CuSQ Hydrogel on Wound Closure in Rats

**Figure 6:**
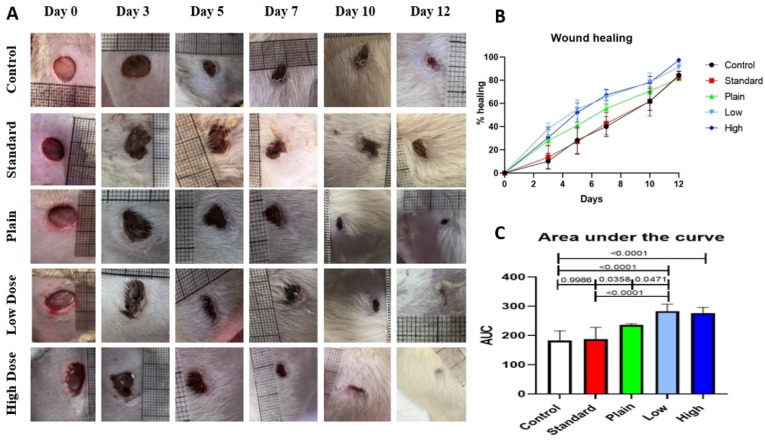
The effect of CuSQ hydrogel on wound healing as compared to standard gentamicin (0.1% garamycin cream). The left panel (**A**) depicts the wounds’ gross appearance from day 0 (the day of wound induction) until day 12. The right panel (**B**) shows the rate of wound healing from day 0 to day 12 (**C**) shows the quantified area under the curve (AUC). Data represented as mean ± SD of at least 8 wounds. When compared to the control and standard, CuSQ at both low and high doses has greater AUC (*p* < 0.0001); the exact *p* values are shown in the graph.

The wound area representative photos of the rats of all the five groups on days 0, 3, 5, 7, 10, and 12 are given in Figure 6A. The healing percentage of wounds of all groups was found to increase in a timely dependent fashion (Figure 6B). Wound healing was markedly faster in both low and high dose CuSQ-treated groups as compared to the control and standard groups as they had highly significant greater area under the curve (AUC) throughout the study period (*p* < 0.0001) (Figure 6C).

### 2.10. Wound Healing Biomarkers

#### 2.10.1. Effect of CuSQ Hydrogel on TGF Beta and MMP-9

**Figure 7:**
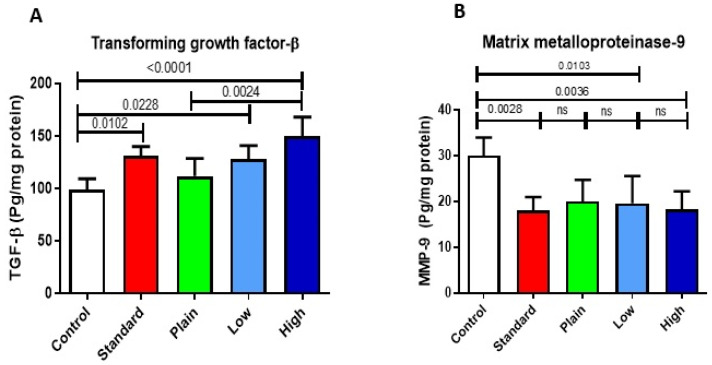
The effect of CuSQ hydrogel on quantitative analysis of the proliferative markers in the healed tissue at the sacrifice day as compared to standard gentamicin (0.1% garamycin cream); matrix metalloproteinase ((**A**), MMP-9), and transforming growth factor beta ((**B**), TGF-β). Data are represented as mean ± SD of at least 6 wounds. The exact *p* values are shown in the graph.

The transforming growth factor beta (TGF-β) was found to be significantly increased in both doses of CuSQ hydrogels compared to the control group; however no significant difference was observed between the plain hydrogel and the control group (Figure 7A). Similarly, both low and high dose of CuSQ hydrogel significantly reduced the MMP-9 level compared to the control group (Figure 7B).

#### 2.10.2. Histopathology and Healing Score

**Figure 8:**
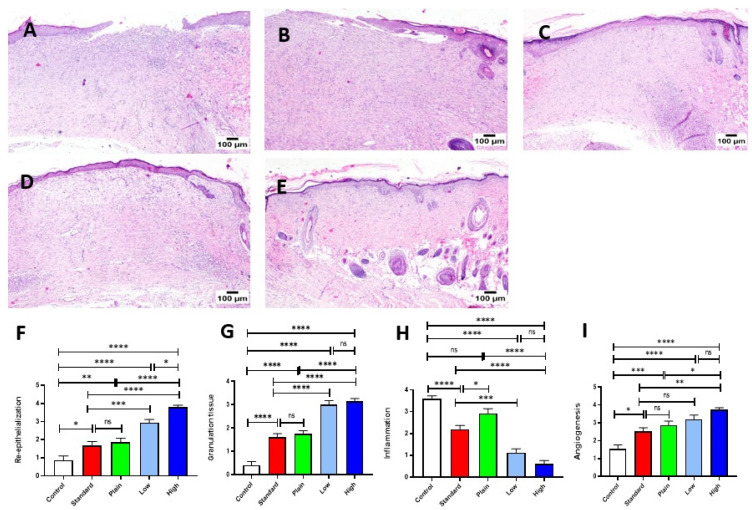
Histopathological examination of the healed tissue. Microscopic images of skin, HE stained (**A**) control group, (**B**) standard gentamicin (0.1% garamycin cream), (**C**) plain, (**D**) low, and (**E**) high. Charts, histopathological evaluation of (**F**) re-epithelization, (**G**) granulation tissue formation, (**H**) inflammation, and (**I**) angiogenesis. Data represented as mean ± SE of at least 5 wounds. **** for *p* < 0.0001, *** for *p* < 0.001, ** for *p* < 0.01, * *p* < 0.05, ^ns^ for non-significant.

Microscopic examination of the wound area from the control group (Figure 8A) revealed signs of incomplete re-epithelization especially toward the center of the wound. Haphazardly arranged granulation tissue was filling the wound area with small areas of organization at the periphery. An intense diffuse inflammatory reaction was noticed at the wound gap. The standard group (Figure 8B) showed a thin line of newly formed epithelium covering the wound surface with the presence of less inflamed granulation tissue filling the wound gap. Concerning the plain group (Figure 8C), re-epithelization and keratinization was observed. The wound gap was filled with organized tissue with numerous newly formed capillaries. Severe inflammatory reactions were noticed at the wound area. Better healing signs were detected in the low dose CuSQ group (Figure 8D), complete and thick epithelial cover was seen over the wound surface and the organized tissue was filling the wound gap completely. Numerous proliferating fibroblasts were observed as well as many newly formed capillaries. Perfect healing and wound closure were observed in the high dose CuSQ group (Figure 8E). Newly developed epithelium with keratinization completely wrapped the wound site. The gap left by the wound was filled with collagen-rich, structured tissue, with little inflammation. As shown in Figure 8F–I, generally, histological score of the wound healing criteria showed significantly improved healing parameters in the high dose group followed by that of the low dose group.

**Figure 9:**
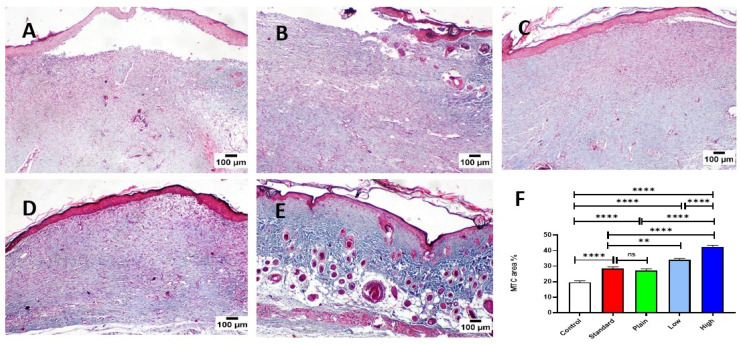
Collagen formation in the healed tissue. Microscopic images of skin, MTC stained (**A**) control group, (**B**) standard gentamicin (0.1% garamycin cream), (**C**) plain, (**D**) low, and (**E**) high (**F**). Quantification of MTC stained collagen (area%). Data represented as mean ± SE of at least 5 wounds. **** for *p* < 0.0001, ** for *p* < 0.01, ^ns^ for non-significant.

The amount of collagen at the wound area was determined using MTC stained sections Figure 9A–F. The lowest amount of collagen was detected in the control group in comparison to the other experimental groups. Both standard and plain groups showed moderate amounts of collagen. Collagen content was significantly increased in both the low dose and high dose CuSQ treated groups in dose-dependent manner.

#### 2.10.3. Immunohistochemistry

##### VEGF Expression

**Figure 10:**
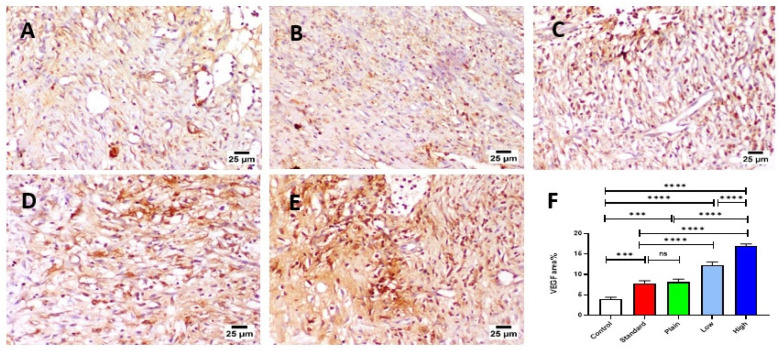
Vascular epithelial growth factor expression on the healed tissue. Microscopic images of skin immunostaining of VEGF. (**A**) Control group, (**B**) standard gentamicin (0.1% garamycin cream), (**C**) plain, (**D**) low, and (**E**) high (**F**) immune expression of VEGF (area%). Data represented as mean ± SE of at least 5 wounds. **** for *p* < 0.0001, *** for *p* < 0.001, ^ns^ for non-significant.

Positive immune staining for VEGF at the wound area in the control group (Figure 10A) was limited in comparison to the other groups. Significantly higher values of VEGF were recorded in both standard (Figure 10B) and plain groups (Figure 10C). Both low-treated (Figure 10D) and high dose treated CuSQ groups (Figure 10E) showed increased VEGF in a dose-dependent manner (Figure 10F).

##### TNF-α Expression

**Figure 11:**
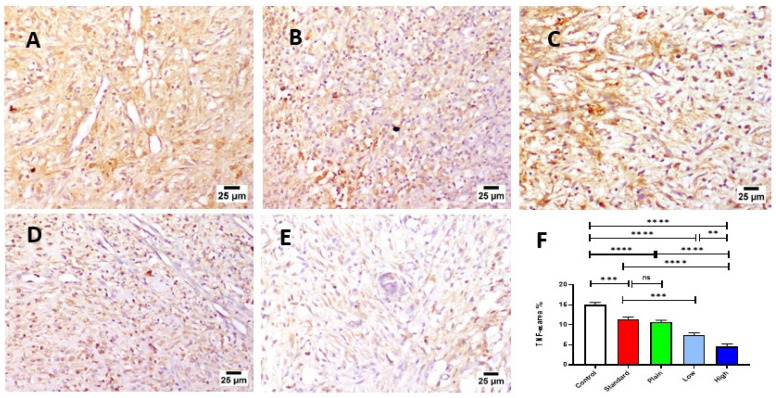
Tumor necrosis factor- α expression on the healed tissue. Microscopic images of skin, immunostaining of TNF-α. (**A**) Control group, (**B**) standard gentamicin (0.1% garamycin cream), (**C**) plain, (**D**) low, and (**E**) high (**F**) immune expression of TNF-α (area%). Data represented as mean ± SE of at least 5 wounds. **** for *p* < 0.0001, *** for *p* < 0.001, ** for *p* < 0.01, ^ns^ for non-significant.

As illustrated in Figure 11, the highest value of TNF-α was recorded in a control group (Figure 11A). Both the standard (Figure 11B) and plain group (Figure 11C) showed significant reduction in TNF-α expression when compared to the control group. The greatest significant decrease in TNF-α expression was recorded in the low dose CuSQ (Figure 11D) and high dose CuSQ (Figure 11E) treated groups, respectively.

## 3. Discussion

Hemostasis, inflammation, proliferation, and remodeling are the four distinct and overlapping stages of wound healing that acute wounds typically go through as they heal [23,24,25]. The process typically begins with an inflammatory response, followed by dermal and epidermal cell migration and proliferation, as well as matrix production, to fill the wound gap and restore the skin barrier [26].

The proliferative phase of wound healing is marked by the presence and abundance of dermal fibroblasts and elevated level of collagen production where diverse factors are implicated as important key player cytokines [27]. The secreted factors include vascular endothelial cell growth factor (VEGF), transforming growth factor (TGF)-β, and matrix metalloproteases (MMPs). The transforming growth factor-beta1 (TGF-1), vascular endothelial growth factor (VEGF), and fibroblast growth factor (FGF) are engaged in the healing process through encouraging cell proliferation by turning on several processes like the development of the extracellular matrix (ECM), re-epithelialization, and angiogenesis [28].

An ideal healing agent should be able to prevent infection, hasten the healing process by modifying growth factors and cytokines as well as be favorable for cell proliferation and matrix deposition, improved re-epithelization and minimal scar formation. It has been previously noted that the coordination of the Schiff base compounds to the metal ions especially copper increased their activity against several pathogenic bacteria [29].

Furthermore, 8-hydroxy quinoline has a strong chelation property and a powerful bactericidal effect especially after chelation with metal ions. In fact, many metal complexes of 8-hydroxy quinoline were screened for antibacterial effects against several strains of bacteria and they showed significantly greater inhibitory activity [30].

Hence, the CuSQ complex has been tested for its antibacterial efficacy against several types of bacteria and was proven to have superior antibacterial activity as compared to gentamicin. Therefore, due to its apparent antibacterial activity together with the powerful efficacy of copper to stimulate angiogenesis and collagen deposition [31], this complex was ideal to be incorporated in a wound healing formulation. Therefore, using nanobiotechnology in conjunction with knowledge of the cellular and subcellular activities that take place during wound healing has enabled us to incorporate this complex into an SLN based hydrogel formulation for the improvement of wound care. To exclude any potential cytotoxicity of copper nanoparticles, the CuSQ SLN were tested for cytotoxicity against human fibroblast cells. To maintain systematic stability, the hydrogel network generally has numerous interactions. Through strong physical crosslinking, such as the production of hydrogen bonds, electrostatic contacts, covalent bonds, hydrophobic interactions, and other physical crosslinks, nanomaterials in hydrogels have significantly increased the mechanical strength of hydrogels. Nanomaterials as SLN can directly transfer antibacterial medications to the wound site during the healing process, enabling them to continue working, lowering the growth of bacteria that are resistant to several antibiotics, and accelerating the healing process [30].

Hydrogel adhesion can be obtained by integrating nanotechnology and hydrogels, for example, by covalent coupling and noncovalent complexes. Additionally, hydrogels can be applied directly to the wound site filling it to enable faster wound healing and the development of capillaries and hair follicles [32].

In fact, small SLN have a large specific surface area, which makes it more likely to stick together when applied to the skin and to have adhesive capabilities through film formation. Water evaporation from the skin is stopped via film development, and CuSQ penetration through human skin is enhanced. The adhesion to the stratum corneum generated by the nanometer-sized particles of SLN boosted CuSQ penetration into viable skin.

Nanohydrogels also reduce the frequent administration issue that is the main drawback of traditional dosage forms, which is helpful in the treatment of chronic wounds. The application potential of nanohydrogels as wound dressings is enhanced by the important role that nanomaterials can play in hydrogels. In addition to enhancing the hydrogel’s mechanical characteristics, water solubility, and cell adhesion, nanotechnology can load different biologically active particles to yield slow release extended drug delivery that has a superior therapeutic effect compared to traditional topical medications. Additionally, a smaller number of medications can function better due to the huge surface area of the nanoparticles, which also somewhat lessens the toxicity of the drugs [33].

Wound contraction is described as a cellular mechanism for organizing the surrounding tissue matrix in order to accelerate healing time by decreasing the quantity of ECM generated [34]. In many ways, wound contraction is advantageous since it can greatly speed up healing by requiring less granulation tissue to replace tissue lost during the healing process [35]. Given the above, measuring wound contraction is a crucial technique for determining how well cutaneous wounds are healing. In the present study, CuSQ hydrogel showed significantly higher wound contraction as compared to the control rats. Although, plain hydrogel showed significantly reduced wound area, the effect was more pronounced in both low and high doses of CuSQ hydrogel. This could be attributed to increased collagen synthesis and better re-epithelization after CuSQ topical application.

Tumor necrosis factor (TNF-α) is a potent inflammatory cytokine released during the inflammatory stage of healing process. Inflammatory cells release TNF-α, which suppresses the production of ECM and activates MMPs. Recent research has shown that inflammatory cytokines have antagonistic effects on TGF-β1. Maintaining tissue homeostasis and ECM deposition may be significantly influenced by the antagonistic connection between TGF-β and TNF-α [36].

TGF-β increases keratinocyte regenerative maturation, attracts fibroblasts to the wound bed, and stimulates fibroblast-induced collagen production [37,38]. It is a crucial cytokine in typical wound healing and fibrosis. Additionally, TGF-β increases the expression of tissue inhibitor of metalloproteinases while downregulating matrix metalloproteinase expression, causing the synthesis of ECM, particularly collagen I, to be stimulated. As a result, it regulates the ECM remodeling and tissue contraction of granulation tissue, two crucial processes in wound healing [36].

Our results showed a significant decrease in TNF-α expression following treatment with our formulation compared to the control rats. The decrease in TNF-α levels on day 12 in the CuSQ-treated rats suggests that it has the potential to reduce the inflammatory response. In addition, the TGF-β level was significantly elevated in CuSQ-treated rats compared to non-treated rats and the standard treated rats which is correlated with increased staining intensity of collagen fibers evidenced by MTC staining, granulation tissue formation as well as re-epithelization in the healed tissues. Many Cu (II) complexes of NSAIDs showed improved anti-inflammatory and antiulcerogenic activities [39]. This was comparable with TNF-α significant decrease in response to the low and high concentrations of CuSQ hydrogel. On comparing the results of both concentrations of CuSQ hydrogels with the plain dosage form it was evident that CuSQ had higher significantly different levels of TGF-β and TNF-α. The mounting levels of TGF-β in the wound of treated rats confirmed the predominant healing ability of the synthesized CuSQ complex. These findings support the study by [40] in copper dressing-treated mice where significant levels of TGF-β gene expression demonstrated a 32-fold increase. Copper influences keratinocyte and fibroblast proliferation, epithelialization, collagen production, extracellular matrix remodeling, and angiogenesis either directly or indirectly. These processes speed up the healing of wounds [40].

These results indicate that CuSQ may act on wound healing via inhibition of inflammation and induction of fibroblast proliferation and granulation tissue formation through stimulating TGF-β [36].

MMP-9 affects angiogenesis by causing proangiogenic cytokines, such as VEGF and TNF-α to become active [41]. Considering that the elevated active MMP-9 expression in skin tissue is linked to the delayed wound healing, our results showed a significantly reduced level of MMP-9 in skin wounds of animals treated by CuSQ hydrogel compared to the control rats which proves the protective effect of our novel formulation. These results are in correlation with the H and E results where significant increase in reepithelization and angiogenesis were detected in rats treated with our formulation confirming the emerging role of the CuSQ hydrogel in modulating the crucial growth factors and cytokines of wound healing machinery leading to accelerating the wound-healing process [42].

The most prevalent, efficient, and persistent signal that is known to promote angiogenesis in wounds is thought to be VEGF. Comparing CuSQ-treated rats to both the control and the standard group, VEGF expression was noticeably higher in the CuSQ-treated group. VEGF expression was significantly elevated in CuSQ-treated rats in comparison to both the control and the standard group. The significant increased VEGF expression levels in healed tissues of the low and high concentrations of synthesized CuSQ complex in a dose-dependent matter owes to the angiogenic effect of Copper II. Copper was reported to be potentially involved in the activation of HIF-1 and VEGF expression [43]. Recent investigations have unmistakably demonstrated that nanoparticles (NPs) represent a crucial therapy platform for skin wounds. Cu is a well-known NP with a lengthy history of direct angiogenesis involvement and antibacterial action. By controlling the expression of 84 genes linked to angiogenesis and wound repair, copper has also been demonstrated to have a possible involvement in the healing of wounds [44].

According to the histology results of our study, the effective role of the CuSQ hydrogel on the wound healing process was clear in the improved healing parameters and perfect wound closure at both low and high doses.

A significant reduction in inflammation in the highly treated group was demonstrated followed by the low-concentration group. In addition, the angiogenic response was promoted as a significant increase in angiogenesis score was noted in both groups and was higher than the standard drug. A significant stimulation in collagen deposition was also observed in a concentration-dependent manner. Copper dependent enzymes such as lysyl oxidase and prolyl 4-hydroxylase play a significant role in collagen synthesis. In addition, enhanced collagen synthesis and deposition may be attributed to enhanced vascularization in CuSQ-treated rats. Furthermore, as there was increased expression of TGF-β and VEGF in our study, the vascularization was subsequently improved. These findings are in alignment with many studies that demonstrated the well-established copper complexes and dressing efficacy in healing acute wounds via promoting neovascularization, increased epithelialization, collagen deposition, and accelerated wound contraction [39,44,45]. Thus, in contrast to control and standard treated groups, CuSQ-treated rats had faster wound healing due to enhanced angiogenesis and collagen synthesis. From the current study, it can be concluded that CuSQ nanoparticle-based hydrogel efficiently promotes various wound healing phases through modulation of different cytokines and growth factors, and hence could be a promising therapeutic modality for the treatment of cutaneous wounds.

## 4. Materials and Methods

### 4.1. Reagents

Ethylene diamine, salicylaldehyde, 8-hydroxy quinoline, sodium hydroxide, copper (II) chloride (CuCl_2_ 2H_2_O) were purchased from Sigma-Aldrich Chemie (Darmstadt, Germany). Organic solvents, including absolute ethanol and dimethyl sulfoxide (DMSO), were provided at reagent grade, and used without purification. Chemicals used for the synthesis of nanoparticles: lecithin, cholesterol, Tween 80, polyvinyl alcohol, and phosphate buffer were purchased from Sigma-Aldrich Chemie (Darmstadt, Germany) and all other chemicals were of analytical grade.

### 4.2. Characterization

Microanalyses of carbon, hydrogen, and nitrogen were performed using a CHNS-932 (LECO) Varo elemental analyzer. FTIR spectrophotometer from Shimadzu (model 8101) In KBr pellets, Fourier transform infrared (FT-IR) spectra between 400 and 4000 cm^−1^ were recorded. A Bruker 400 MHz spectrometer was used to record the ^1^H and ^13^C NMR spectra in DMSO-d_6_, with tetra methyl silane (TMS) serving as an internal standard. The electron ionization method was utilized to record mass spectra at 70 eV on an HP MS-5988 GS-MS. UV-visible spectra were recorded using a Shimadzu UV mini-1240 spectrophotometer. The molar conductivity of 10^−3^ M solutions of the CuSQ complex in DMF was measured using a Jenway 4010 conductivity meter [45].

### 4.3. Synthesis of Schiff Base (Salen)

The method of Schiff base synthesis was previously discussed [45]. The analytical and spectral data of Salen ligand are: yield 96%; M. p. 127 °C; FTIR (KBr, cm^−1^): 3292 (OH), 3049–3007 (CH)_arom_., 2899–2867 (CH)_aliph_, 1608 (C=N), 1217 (C-C); ^1^H NMR (DMSO-*d*_6_/D_2_O, 400 MHz): 3.87 (t, 4H, N-CH_2_-CH_2_-N), 6.69–7.37 (m, 8H, Ar-H), 8.54 (s, 2H, 2 N=CH), 13.3 (br s, 2H, 2 ArOH); ^13^C NMR (100 MHz, DMSO-*d*_6_): 58.68, 116.99, 119.00, 132.22, 132.94, 161.19, and 167.28.

### 4.4. Synthesis of Mixed Complex (CuSQ)

**Scheme 1:**
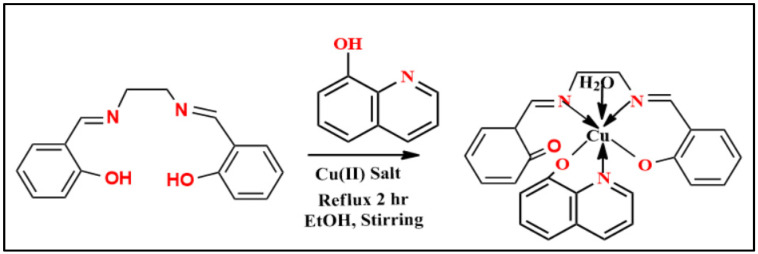
Synthesis pathway for CuSQ mixed Salen ligand complex with 8-hydroxy quinolone.

Scheme 1 shows the synthesis pathway for CuSQ mixed Salen ligand complex with 8-hydroxy quinoline. The yields, melting points, conductance, magnetic moments, and elemental analysis results for the mixed ligand complex are shown in Table 1 [11].

### 4.5. Antimicrobial Potency

The antimicrobial activity of the Cu (II) complex was evaluated using two Gram-positive bacteria (*Bacillus subtilis* (+ve) and *Staphylococcus aureus* (+ve)) and two Gram-negative bacteria (*Escherichia coli* (-ve) and *Proteus vulgaris* (-ve)) by the agar diffusion test. The compound under investigation was dissolved in DMSO and paper discs were impregnated with the solution. The disks were dried and set in agar plates containing the microorganisms [45]. Then, the plates were incubated for 25–33 h at 24 ± 2 °C and the inhibition zones, i.e., those where the concentration of the compound exceeds the minimum inhibitory concentration (MIC), were accurately evaluated. Gentamicin as an antibacterial agent was used for comparison.

### 4.6. Synthesis of CuSQ Solid Lipid Nanoparticles (SLN)

Solid lipid nanoparticles of CuSQ were prepared by w/o/w type double emulsification method as shown in Table 6. The preparation method was previously described elsewhere [46].

### 4.7. Characterization of CuSQ-Loaded SLNs

#### 4.7.1. Scanning Electron Microscopy (SEM)

The surface morphology of CuSQ SLNs was observed by scanning electron microscope (SEM). The image was scanned at an acceleration voltage of 20 kV with a chamber pressure of 0.8 mmHg [47].

#### 4.7.2. Particle Size Analysis, Polydispersity Index and Zeta Potential

CuSQ SLNs dispersions were characterized for average particle size (z-average size) using Laser diffraction (Malvern Mastersizer 2000 SM, Malvern Instruments Corp) with beam length 2.40 mm, range lens of 300 RF mm, and at 14.4% obscuration. Polydispersity (PDI) describes the degree of non-uniformity of a size distribution of particles. This index is dimensionless and scaled such that values smaller than 0.05 are mainly seen with highly monodisperse standards. PDI values bigger than 0.7 indicate that the sample has a very broad particle size distribution. Zeta potential (surface charge) was determined by measuring the electrophoretic mobility of nanoparticles using Malvern Mastersizer [46].

#### 4.7.3. Entrapment Efficiency

The entrapment efficiency was calculated by using 10 mL of solid lipid nanoparticles dissolved in 20 mL of ethyl alcohol and the solution was centrifuged at 12,000 rpm. The supernatant fluid was collected and passed through a membrane filter. The quantity of drug in the solution was measured by ultraviolet spectroscopy at 280 nm [46].

### 4.8. Cytotoxicity of CuSQ Loaded SLNs

#### 4.8.1. Cell Culture

HSF: human skin fibroblast was obtained from Nawah Scientific Inc., (Mokatam, Cairo, Egypt). Cells were maintained in DMEM media supplemented with 100 mg/mL of streptomycin, 100 units/mL of penicillin and 10% of heat-inactivated fetal bovine serum in humidified, 5% (*v*/*v*) CO_2_ atmosphere at 37 °C.

#### 4.8.2. Cytotoxicity Assay

Cell viability was assessed by SRB assay using a BMG LABTECH^®^-FLUO star Omega microplate reader (Ortenberg, Germany) [48,49].

### 4.9. Preparation of CuSQ Nanoparticles Hydrogel

The hydrogel was prepared by dissolving the hydrophilic polymers carbopol 940 in a 0.1N NaOH solution used as a cross-linking agent with addition of water using magnetic stirrer (500 rpm). The colloidal dispersion of CuSQ solid lipid nanostructures were added under magnetic stirring. 1% *v*/*v* isopropyl myristate and 0.25% *w*/*v* benzalkonium chloride were added (Table 7) [50].

#### 4.9.1. Evaluation of Hydrogel Physical Characteristics

The prepared hydrogel formulations were inspected visually for their pH, color, homogeneity, consistency, grittiness, texture, and phase separation [50].

#### 4.9.2. Determination of pH

The pH of hydrogel formulations was determined by digital pH meter. One gram of gel was dissolved in 25 mL of distilled water and the electrode was then dipped into gel formulation for 30 min until constant reading obtained and constant reading was noted. The measurement of pH for each formulation was carried out in triplicate and average values were calculated [50].

#### 4.9.3. Washability

Formulations were applied on the skin and then ease and extent of washing with water were checked manually [50].

#### 4.9.4. In Vitro Drug Dissolution of Hydrogel

Franz diffusion cell with cellophane membrane was used for in vitro drug release studies of loaded hydrogel. The procedure was carried out according to the method of Singh et al. [51]. Samples were analyzed by using a UV spectrophotometer at 280 nm. Experiments were carried out in triplicate to ensure accuracy.

### 4.10. In Vivo Assessment of the Wound Healing Activity

#### 4.10.1. Animals

A total number of 20 male Wistar albino rats (120–150 g) were utilized for wound healing experimental study. They were supplied by the Modern veterinary office at Cairo. Animals were kept for 1-week acclimatization at the animal house of October University for Modern Sciences and Arts (MSA). The animals were housed in clean cages and maintained in 12:12 h dark light cycle in standard room temperature of 22 ± 3 °C. They had ad libitum access to drinking water and food. Calculation of the sample size (*n* = 8) was carried out using the G * Power 3.1.9 software, the statistical power used was 80% and alpha value = 0.05. All animals’ procedures were carried out according to the ethical guidelines of MSA ethical committee (PT6/EC6/2020F).

#### 4.10.2. Excision Wound

Excision wounds were created according to the method of Mukherjee et al. [52] where animals were anesthetized by injection of a mixture of xylazine (7 mg/kg) and ketamine (70 mg/kg) intraperitoneally. After that, their dorsal hair was shaved and cleaned with 70% ethanol and using a 1 cm biopsy punch 2 circular open excision wounds (1 cm^2^) were made per rat where the full thickness of the back skin was cut out. The wounds were then cleaned with saline and the animals were housed individually.

#### 4.10.3. Experimental Design

Animals were randomly allocated into 5 groups (4 rats/group). Group 1 served as control group and the wounds of these animals were left without treatment. Group II served as the standard group were animals received standard gentamicin (0.1% garamycin cream). Group III received the plain hydrogel. Groups IV received low dose CuSQ hydrogel (0.05 ug/mL). Groups V received high dose CuSQ hydrogel (0.2 ug/mL). All treatments were applied every other day.

#### 4.10.4. Estimation of the Rate of Wound Healing

Progressive changes in wound healing were assessed quantitatively using image J 1.52 software. The wound area was calculated using scaled pictures of the wounds on days 0, 3, 5, 7, 10, and 12. Percentage healing was calculated using the following equation, where *n*= number of days (3, 5, 7, 10, or 12) that the healed area was read.

$$\mathrm{Percentage}\;\mathrm{healing}\;(\%)=\frac{\mathrm{wound}\;\mathrm{area}\;\mathrm{at}\;\mathrm{day}\;0-\mathrm{wound}\;\mathrm{area}\;\mathrm{in}\;\mathrm{day}\;n}{\mathrm{wound}\;\mathrm{area}\;\mathrm{at}\;\mathrm{day}\;0}\;\times \;100$$

### 4.11. Wound Healing Markers

#### 4.11.1. Enzyme-Linked Immunosorbent Assay (ELISA)

Both transforming growth factor β1 (TGF-β1) (Cusabio, CSB-E04727r) and matrix metalloproteinase-9 (MMP-9) (Mybiosource, MBS722532) were assessed in the tissues of healed wounds using ELISA kits’ instructions.

#### 4.11.2. Histopathological Examination

Skin tissue samples from the wound area were kept in 10% neutral buffered formalin. Specimen were routinely processed and stained with hematoxylin and eosin (HE) for light microscopy [53]. Microscopic wound healing criteria were evaluated including re-epithelization, granulation tissue formation, inflammation, and angiogenesis as described in [54] with modification of inflammation score as it was described in ascending score with increased severity. Masson trichrome stain (MTC) was used for evaluation of collagen content at the wound area.

#### 4.11.3. Immunohistochemistry

Five µm tissue sections were cut into adhesive slides, deparaffinized, rehydrated, and subjected to the epitope retrieval method by heat, endogenous peroxidase blocking, and protein blocking. Primary mouse monoclonal anti VEGF and anti-TNF-α (at a dilution of 1:150) for 12 h at 4 °C. After washing, secondary HRP-labeled antibody was applied at room temperature for 2 h. DAB-Substrate kit was used to develop the color. Control negative slides were obtained by deletion of primary antibodies. Positive expression was quantified as area % using CellSens dimensions (Olympus software).

### 4.12. Statistical Analysis

Statistical analysis was performed using the GraphPad Prism version 8.1.2 software utilizing analysis of variance (ANOVA) followed by Tukey’s multiple comparisons test or the paired t-test, where appropriate, and statistical significance was set at *p* < 0.05. Significance was presented as: **** for *p* < 0.0001, *** for *p* < 0.001, ** for *p* < 0.01, and * for *p* < 0.05.

## 5. Conclusions

In the present study CuSQ mixed ligand complex was synthesized and characterized by several analytical and spectroscopic tools. Results of antimicrobial study have demonstrated a powerful antibacterial effect that was more potent than the standard gentamicin drug. In vivo study of CuSQ SLN-loaded hydrogel showed more powerful wound healing activity compared to gentamicin. CuSQ hydrogel has shown prominent anti-inflammatory and pro-angiogenic effects and enhanced tissue remodeling and collagen deposition. It could thus be concluded that CuSQ nanoparticles-based hydrogel efficiently promotes various wound healing phases and could be a promising nano formulation for cutaneous wound healing acceleration.

## Figures and Tables

**Table 1:** Analytical data of Salen and its CuSQ mixed ligand complex of 8-hydroxy quinolone.

Molecular Formula	Molecular Weight	Color Yield%	Conductivity µs	M. P. °C	µ_eff_µ B. M	Found (Cal.) %			
						C	H	N	M
Salen C_16_H_16_N_2_O_2_	268.31	Yellow 95	0.830	127	0.58	71.32	5.95	10.28	-
Cu (S) (Q) H_2_O C_25_H_24_CuN_3_O_4_	494.02	Yellowish green 77	24.10	264	1.50	60.38	4.62	8.38	12.73

**Table 2:** FTIR Spectral data of Salen and its CuSQ complex of 8-hydroxy quinolone.

Compound	ν (OH)	ν (NH)	ν (CH)_arom_	ν (CH)_aliph_	ν (C=O)	ν (C=N)	ν (C-O)	ν (H_2_O) _Coordinated_	ν (M-O)	ν (M-N)
**Salen**	3292	-	3049–3007	2899–2867	-	1608	1247	-	-	-
**CuSQ**	3241	3200	3050	2968–2862	1740	1644	1213	945	516	442

**Table 3:** Electronic spectra of Salen ligand and its CuSQ complex.

Compounds	λ _max_ (nm)	Assignment
S	280	π—π *
	320	*n*—π *
	409	*n*—π *
CuSQ	280	π—π *
	380	*n*—π *

**Table 4:** Antibacterial bioassay of Salen ligand and its CuSQ complex against different strains of bacteria.

Compound	*S. aureus* (+ve)	*B. subtilis* (+ve)	*E. coli* (−ve)	*P*. *vulgaris* (−ve)
S	14	13	12	15
Q	16	14	13	16
CuS	25	20	13	22
Gentamicin	24	26	30	25
CuSQ	35	27	18	33

**Table 5:** Particle size, PDI, zeta potential, and entrapment efficiency (EE%) of the formulations of different CuSQ—loaded solid lipid nanoparticles (SLNs).

	Particle Size (nm)	PDI (%)	Zeta Potential (mv)	EE (%)
**F1**	111 ± 0.26	0.52 ± 0.11	−11.8 ± 0.13	88 ± 0.12
**F2**	136 ± 0.32	0.43 ± 0.12	−40 ± 0.23	85 ± 0.11

**Table 6:** Composition for formulations of different CuSQ-loaded solid lipid nanoparticles (SLNs).

*Materials*	*F1*	*F2*
CuSQ (mg)	25	50
Soy Lecithin (mg)	10	10
Cholesterol (mg)	30	30
Dimethyl sulphoxide (DMSO) (mL)	10	10
Tween 80 (mL)	2	2
2% *w*/*v* of polyvinyl alcohol (mL)	2	2
H_2_O (mL)	30	30

**Table 7:** Formulation of both concentrations of CuSQ SLNs hydrogel.

Ingredients	Formula with Low Concentration (F1)	Formula with High Concentration (F2)
Carbopol 940	0.7 mg	0.7 mg
Copper shiff base dispersions	5 mL	15 mL
Water	15 mL	15 mL
0.1 NaoH	2 mL	2 mL
Isopropyl myristate	1%	1%
Benzalkonium chloride	0.25	0.25

## Data Availability

Data is contained within the article.
